# Anatomical breast imaging-derived parameters do not provide incremental information in prediction of nonvisualization of sentinel lymph nodes on lymphoscintigraphy

**DOI:** 10.1097/MNM.0000000000001585

**Published:** 2022-05-18

**Authors:** Youssef Chahid, Hein J. Verberne, Edwin Poel, N. Harry Hendrikse, Jan Booij

**Affiliations:** Departments of a Radiology and Nuclear Medicine; b Clinical Pharmacy, Amsterdam UMC, location AMC; c Department of Radiology and Nuclear Medicine, Amsterdam UMC, location VUmc, Amsterdam, The Netherlands

**Keywords:** breast cancer, breast MRI, lymphoscintigraphy, mammography, nonvisualization, sentinel lymph node

## Abstract

**Objective:**

Accurate sentinel lymph node (SLN) staging is essential for both prognosis and treatment in patients with breast cancer. However, the preoperative lymphoscintigraphy may fail to visualize the SLN. The aim of this retrospective study was to investigate whether parameters derived from anatomical breast imaging can predict SLN nonvisualization on lymphoscintigraphy.

**Methods:**

For this retrospective study, all data of mammography, breast MRI, and lymphoscintigraphy of SLN procedures from January 2016 to April 2021 were collected and reviewed from the Amsterdam UMC database.

**Results:**

A total of 758 breast cancer patients were included in this study. SLN nonvisualization on planar lymphoscintigraphy at 2-h postinjection (pi) was 29.7% and was reduced after a second injection to 7.5% at late lymphoscintigraphy 4-h pi. Multivariable analysis showed that age ≥ 70 years (*P* = 0.019; OR, 1.82; 95% CI, 1.10–3.01), BMI ≥ 30 kg/m^2^ (*P* = 0.031; OR, 1.59; 95% CI, 1.04–2.43), and nonpalpable tumors (*P* = 0.034; OR, 1.54; 95% CI, 1.03–2.04) were independent predictors of SLN nonvisualization. Differences in tumor size, Breast Imaging-Reporting and Data System classification, or breast density were not significantly associated with SLN nonvisualization.

**Conclusion:**

This study shows that, by using a multivariable analysis, risk factors for SLN nonvisualization in breast cancer patients during preoperative lymphoscintigraphy at 2-h pi are age ≥ 70 years, BMI ≥ 30 kg/m^2^, and nonpalpable tumors. Parameters derived from mammography or breast MRI, however, are not useful to predict SLN nonvisualization on lymphoscintigraphy.

## Introduction

Accurate sentinel lymph node (SLN) staging is essential for both prognosis and treatment in patients with breast cancer selected to undergo an SLN procedure [1]. However, preoperative lymphoscintigraphy may fail to visualize the SLN. In the literature, reported rates of SLN nonvisualization vary between 2 and 28% [2–7]. After investigation of a large dataset of breast cancer patients (*n* = 1462), we recently reported that age ≥ 70 years [*P* < 0.001; odds ratio (OR), 2.27; 95% confidence interval (CI), 1.46–3.53], BMI ≥ 30 kg/m^2^ (*P* = 0.031; OR, 1.48; 95% CI, 1.04–2.12), and nonpalpable tumors (*P* = 0.004; OR, 1.54; 95% CI, 1.15–2.07) were independent predictors of SLN nonvisualization on lymphoscintigraphy at 2-h postinjection (pi) [8]. These findings were in accordance with findings of some previous studies [2–7].

Shortly after our publication, however, Quak *et al*. [9] showed an interesting association between breast density and SLN nonvisualization. They found that breasts with fatty or scattered fibroglandular densities were strongly associated with higher age (*P* < 0.001), higher BMI (*P* < 0.001), and nonvisualization (*P* = 0.042). However, due to a lack of a multivariable analysis, it was not possible to assess the influence of breast density in relation to other risk factors for SLN nonvisualization [10].

Therefore, the purpose of this retrospective study was to reproduce the claim that breast density is an independent predictor of SLN nonvisualization on lymphoscintigraphy.

## Methods

### Patient population and data extraction

Selected patients of this study were part of a single-center retrospective study investigating lymphoscintigraphy data of SLN procedures for risk factors for nonvisualization [8]. This study was approved by the local Medical Ethics Review Committee. Data of SLN procedures were collected from January 2016 to April 2021 and reviewed. The following data were collected from the Amsterdam UMC, location AMC, electronic health records database: age (divided into three categories: <50 years, 50–70 years, and ≥70 years) [6], BMI (divided into three categories: <25 kg/m^2^, 25–30 kg/m^2^, and ≥30 kg/m^2^) [5], tumor palpability (divided into two categories: palpable and nonpalpable tumors) [6], and tumor location (divided into two categories: lateral and medial/central) [6], and tumor size [divided into three categories: <20 mm (T-stage 1), 20–50 mm (T-stage 2), and >50 mm (T-stage 3)] [6]. The following characteristics were divided based on the distribution of our data: breast imaging-reporting and data system (BI-RADS) classification (divided into classification ≤5 and classification 6) and breast density categories [divided into ≤50% fibroglandular density (categories A or B) and >50% fibroglandular density (categories C or D)] [11].

### Lymphoscintigraphy imaging protocol

Technetium-99m-labeled albumin nanocolloid (from January 2016 to February 2019: Nanocoll, GE Healthcare, Eindhoven, The Netherlands; from March 2019 to April 2021: Nanoscan, Radiopharmacy, Budaörs, Hungary) was administrated via an intratumoral injection, by a resident or an experienced nuclear medicine physician, either by palpation in palpable tumors or ultrasound-guided in nonpalpable tumors. An injected dose of approximately 120-MBq technetium-99m-labeled albumin nanocolloid in a volume of 0.25 ml was administered in the afternoon of the day prior to surgery. Planar lymphoscintigraphy was performed at 15-min pi and 2-h pi. If planar lymphoscintigraphy showed SLN nonvisualization at 2-h pi, single photon emission computed tomography/computed tomography (SPECT/CT) imaging or a second periareolar injection of 120 MBq, followed by repeated planar lymphoscintigraphy, and sometimes additional SPECT/CT imaging, 2-h later (i.e. 4-h pi), were performed. As nonvisualization at 2-h pi is an important decision moment for further diagnostic intervention, we focused our analysis on this time point. Focal accumulations in at least one axillar lymph node were defined as SLN. SLN nonvisualization was clinically classified as nonvisualization when no SLN was visualized on routine clinical lymphoscintigraphy, as earlier described [8].

### Breast imaging protocol

Breast imaging was visually assessed by an experienced radiologist in accordance with the fifth edition of BI-RADS lexicon on mammography and/or breast MRI. All available data of BI-RADS classification (0–6), breast density (categories A–D), and tumor diameter were collected from the electronic health records.

### Statistical analysis

Patient and tumor characteristics were evaluated using descriptive statistics. Univariate logistic-regression models were used to examine the relationships between the different characteristics and SLN nonvisualization at 2-h pi. Pearson Chi-square exact test was used for categorical variables, and the Mantel–Haenszel exact test was used for ordinal variables. Variables with a *P*-value less than 10% in the univariate analysis were included for the multivariable logistic-regression models. All statistical tests were two-tailed, and a *P*-value less than 5% was considered statistically significant. ORs of significant risk factors are presented with calculation of 95% CI. All analysis were performed with IBM SPSS Statistics (version 26, IBM Corp, Armonk, New York, USA).

## Results

### Preoperative lymphoscintigraphy

A total of 758 breast cancer patients were enrolled in this study. Mean patient age was 59.8 years (SD, 12.0 years), and the mean BMI was 27.7 kg/m^2^ (SD, 5.6 kg/m^2^). Preoperatively, the SLN was not visualized on planar lymphoscintigraphy at 2-h pi in 29.7% (225/758) of the SLN procedures. The nonvisualization of the SLN after SPECT/CT imaging or a second periareolar injection was further reduced to 7.5% (57/758) at late lymphoscintigraphy 4-h pi. A total of 299 patients had breast density categories A (30/299) or B (269/299), and 170 patients had breast density category C (128/170) or category D (42/170).

### Risk factors of sentinel lymph node nonvisualization

Table 1 presents the number of SLN procedures with nonvisualization at planar lymphoscintigraphy at 2-h pi. The multivariable analysis showed that age ≥ 70 years (*P* = 0.019; OR, 1.82; 95% CI, 1.10–3.01), BMI ≥ 30 kg/m^2^ (*P* = 0.031; OR, 1.59; 95% CI, 1.04–2.43), and nonpalpable tumors (*P* = 0.034; OR, 1.54; 95% CI, 1.03–2.04) were independent predictors of SLN nonvisualization on lymphoscintigraphy. Differences in tumor size and BI-RADS classification did not lead to a significant increased risk for SLN nonvisualization at 2-h pi.

**Table 1.** Results of multivariable analysis for risk factors of sentinel lymph node nonvisualization on lymphoscintigraphy at 2-h postinjection of the radiotracer

Characteristics	*N*	N of nonvisualization (%)	Univariate analysis	Multivariable analysis	
			*P*-value	Adjusted OR (95% CI)	*P*-value
Age (years)					
<50	166	40 (24.1)	0.001^a^	1	
50–70	417	114 (27.3)		1.00 (0.65–1.56)	0.985
≥70	175	71 (40.6)		1.82 (1.10–3.01)	0.019
BMI (kg/m^2^)					
<25	236	59 (25.0)	0.007^a^	1	
25–30	223	72 (32.3)		1.37 (0.90–2.07)	0.138
≥30	197	73 (37.1)		1.59 (1.04–2.43)	0.031
Unknown	102				
Tumor palpability					
Palpable	447	109 (24.4)	<0.001^b^	1	
Nonpalpable	311	116 (37.3)		1.54 (1.03–2.04)	0.034
Tumor size (mm)					
<20	541	159 (29.4)	0.969^a^		
20–50	143	42 (29.4)			
>50	14	4 (28.6)			
Unknown	60				
BI-RADS classification					
≤5	187	50 (26.7)	0.218^b^		
6	442	140 (31.7)			
Unknown	129				
Breast density category					
A or B	299	95 (31.8)	0.030^b^		
C or D	170	38 (22.4)			
Unknown	289				

BI-RADS, breast imaging-reporting and data system; CI, confidence interval; OR, odds ratio.

a Mantel–Haenszel exact test for ordinal variables.

b Pearson Chi-square exact test for categorical variables.

### Breast density

The association of breast density categories, age, and BMI is shown in Table 2. The breast density category A or B was significant associated with a higher age (*P* < 0.001) and BMI (*P* < 0.001). The univariate analysis showed that breasts with 50% or less fibroglandular tissue was a predictor of SLN nonvisualization on lymphoscintigraphy at 2-h pi (Table 1; *P* = 0.030). When combined with age, BMI, and tumor palpability in the multivariate analysis, however, breast density was not significantly associated with SLN nonvisualization on lymphoscintigraphy at 2-h pi (*P* = 0.234).

**Table 2.** Comparison of age and BMI of breast density categories

Characteristics	*N*	Breast density categories A or B (%)	Breast density categories C or D (%)	*P*-value
Age (years)				<0.001^a^
<50	135	62 (45.9)	73 (54.1)	
50–70	241	159 (66.0)	82 (34.0)	
≥70	93	78 (83.9)	15 (16.1)	
Unknown	289			
BMI (kg/m^2^)				<0.001^a^
<25	168	82 (48.8)	86 (51.2)	
25–30	145	96 (66.2)	49 (33.8)	
≥30	106	96 (90.6)	10 (9.4)	
Unknown	339			

a Mantel–Haenszel exact test for ordinal variables.

## Discussion

We found that age at least 70 years, BMI of at least 30 kg/m^2^, and nonpalpable tumors were independent predictors of SLN nonvisualization on lymphoscintigraphy at 2-h pi in breast cancer patients. These risk factors were recently discussed in detail in our previous publication [8].

To the best of our knowledge, this study is the first to examine the influence of BI-RADS classification on SLN nonvisualization. We also attempted to reproduce the claim that breast density is an independent predictor of SLN nonvisualization on lymphoscintigraphy [9]. We here showed that BI-RADS classification, breast density, and tumor size were not significantly associated with SLN nonvisualization at 2-h pi.

Breasts with fatty or scattered fibroglandular densities are less reported as a possible risk factor for SLN nonvisualization on lymphoscintigraphy [9]. We found that breast cancer patients with less than 50% fibroglandular density are strongly correlated with higher ages and higher BMI values. These finding are in line with literature [9,12]. We attempted to reproduce the claim of Quak *et al*. [9] that breast density is an independent predictor of SLN nonvisualization. We did find a significant effect of breast density categories on SLN nonvisualization at 2-h pi in an univariate analysis; however, this effect was NS in our multivariable model with predictors as age, BMI, and tumor palpability. The explanation for this phenomenon is that our multivariable model includes both age and BMI, which are strongly correlated with breast density. We conclude that information on breast density categories does not improve the already-known multivariable model with age, BMI, and tumor palpability on SLN nonvisualization.

Tumor size, or T-stage, is a well-studied and controversial parameter as risk factor for SLN nonvisualization on lymphoscintigraphy in breast cancer patients. Although two different studies reported in univariate analyses a significant correlation between tumor size > 20 mm and SLN nonvisualization lymphoscintigraphy in breast cancer patients, Hellingman *et al*. [6] showed in a multivariable model an equal correlation between tumor size and SLN nonvisualization [6,7,13]. Despite this fact, we and the vast majority of other research groups were unable to find any significant effect of tumor size on SLN nonvisualization [2–5,14–19].

To the best of our knowledge, this study is the first to examine the influence of BI-RADS classification on SLN nonvisualization. BI-RADS classification is a risk assessment tool, developed by American College of Radiology, that provides an approximate risk of malignancy to a lesion from zero to at least 95% [20]. Despite the large amount of BI-RADS classification data, there was no significant association with SLN nonvisualization on lymphoscintigraphy at 2-h pi in breast cancer patients.

One of the strengths of this study is the large number of breast cancer patients with lymphoscintigraphy data, which offered the opportunity to perform a multivariable analysis. However, this study has some limitations that need to be addressed. Because not all patients received breast imaging (i.e. mammography or breast MRI) before the SLN lymphoscintigraphy, we were unable to collect all data of BI-RADS classification, breast density, and tumor diameter from the electronic health records. Despite this limitation, the number of the patients in whom these parameters were registered had sufficient statistical power to examine the effect on SLN nonvisualization. Other limitations were that tumor grade and the number of positive lymph nodes were not available. These factors could be confounders, since some studies have indications that these factors are possible associated with SLN nonvisualization [2,3,13,15,16].

### Conclusion

This study shows that, by using a multivariable analysis, risk factors for SLN nonvisualization in breast cancer patients during preoperative lymphoscintigraphy at 2-h pi are age at least 70 years, BMI at least 30 kg/m^2^, and nonpalpable tumors. Parameters derived from mammography or breast MRI, however, do not provide incremental information in the prediction of SLN nonvisualization on lymphoscintigraphy. The findings of this study resulted in a critical evaluation of our SLN procedures to reduce SLN nonvisualization in patients at high risk for nonvisualization (i.e. age ≥ 70 years, BMI ≥ 30 kg/m^2^, and nonpalpable tumors).

## Acknowledgements

### Conflicts of interest

There are no conflicts of interest.
